# Development and characterization of a tamoxifen-resistant breast carcinoma xenograft

**DOI:** 10.1054/bjoc.2000.1156

**Published:** 2000-06

**Authors:** H Naundorf, M Becker, A E Lykkesfeldt, B Elbe, C Neumann, B Büttner, I Fichtner

**Affiliations:** Max-Delbrück-Center for Molecular Medicine, Robert-Rössle-Strasse 10, Berlin, 13092, Germany; Institute of Cancer Biology, Danish Cancer Society, Strandboulevarden 49, Copenhagen, DK-2100, Denmark

**Keywords:** tamoxifen resistance, breast cancer, xenograft, oestrogen receptor, cathepsin D gene, pS2 gene

## Abstract

A human tamoxifen-resistant mammary carcinoma, MaCa 3366/TAM, originating from a sensitive parental xenograft 3366 was successfully established by treatment of tumour-bearing nude mice with 1–50 mg kg^−1^tamoxifen for 3 years during routine passaging. Both tumours did not differ significantly in OR- and PR-positivity, however, when compared with the sensitive tumour line, the mean OR content of the TAM-resistant subline is slightly lower. An OR-upregulation following withdrawal of oestradiol treatment was observed in the parental tumours but not in the resistant xenografts. Following long-term treatment with tamoxifen, the histological pattern of the breast carcinoma changed. The more differentiated structures being apparent after treatment with 17β-oestradiol in the original 3366 tumour were not induced in the resistant line. Tamoxifen failed to induce a tumour growth inhibition in comparison to the tamoxifen-sensitive line. The pure anti-oestrogen, ICI 182 780, revealed cross-resistance. Sequence analysis of the hormone-binding domain of the OR of both lines showed no differences, suggesting that either mutations in other regions of the OR are involved in the TAM-resistance phenotype or that mechanisms outside of this protein induced this phenotype. Oestrogen and anti-oestrogen regulate pS2 and cathepsin D expression in 3366 tumours as in the human breast cancer cell line MCF-7. The resistant 3366/TAM tumours have lost this regulation. The established breast cancer xenografts 3366 and 3366/TAM offer the possibility of investigating mechanisms of anti-oestrogen resistance in an in vivo situation. They can be used to test novel approaches to prevent, or to overcome, this resistance in a clinically related manner. © 2000 Cancer Research Campaign


*British Journal of Cancer* (2000) **82**(11), 1844–1850
© 2000 Cancer Research Campaign
DOI: 10.1054/ bjoc.2000.1156, available online at http://www.idealibrary.com on IDEAL®

### Development and characterization of a tamoxifen-resistant breast carcinoma xenograft

**H Naundorf[1], M Becker[1], AE Lykkesfeldt[2], B Elbe[1], C Neumann[1], B Büttner[1] and I Fichtner[1]**

[1]Max-Delbrück-Center for Molecular Medicine, Robert-Rössle-Strasse 10, 13092 Berlin, Germany; [2]Institute of Cancer Biology, Danish Cancer Society, Strandboulevarden 49, DK-2100, Copenhagen, Denmark

**Summary** A human tamoxifen-resistant mammary carcinoma, MaCa 3366/TAM, originating from a sensitive parental xenograft 3366 was successfully established by treatment of tumour-bearing nude mice with 1–50 mg $kg^{-1}$ tamoxifen for 3 years during routine passaging. Both tumours did not differ significantly in OR- and PR-positivity, however, when compared with the sensitive tumour line, the mean OR content of the TAM-resistant subline is slightly lower. An OR-upregulation following withdrawal of oestradiol treatment was observed in the parental tumours but not in the resistant xenografts. Following long-term treatment with tamoxifen, the histological pattern of the breast carcinoma changed. The more differentiated structures being apparent after treatment with 17β-oestradiol in the original 3366 tumour were not induced in the resistant line. Tamoxifen failed to induce a tumour growth inhibition in comparison to the tamoxifen-sensitive line. The pure anti-oestrogen, ICI 182 780, revealed cross-resistance. Sequence analysis of the hormone-binding domain of the OR of both lines showed no differences, suggesting that either mutations in other regions of the OR are involved in the TAM-resistance phenotype or that mechanisms outside of this protein induced this phenotype. Oestrogen and anti-oestrogen regulate pS2 and cathepsin D expression in 3366 tumours as in the human breast cancer cell line MCF-7. The resistant 3366/TAM tumours have lost this regulation. The established breast cancer xenografts 3366 and 3366/TAM offer the possibility of investigating mechanisms of anti-oestrogen resistance in an in vivo situation. They can be used to test novel approaches to prevent, or to overcome, this resistance in a clinically related manner. © 2000 Cancer Research Campaign

**Keywords**: tamoxifen resistance; breast cancer; xenograft; oestrogen receptor; cathepsin D gene; pS2 gene

Breast cancer is the most frequent malignancy of the female population in the Western world. Prognosis of disease and treatment strategies are mainly determined by the presence of hormone receptors in tumour tissues. At the time of diagnosis about 60% of breast carcinomas express either the oestrogen receptor (OR) or both the oestrogen and the progesterone receptor (PR). Upon signs of dissemination, these malignancies are treated with an endocrinological therapy. For this purpose, mainly the anti-oestrogen tamoxifen (TAM) is primarily used. Unfortunately, about 1/3 of hormone receptor-positive breast cancers initially fail to respond to a tamoxifen therapy, and a large number of originally sensitive tumours develop resistance during several months of treatment (Early Breast Cancer Trial Group 1992). Different hypotheses have been discussed to explain the development of TAM-resistance:

– Degradation of TAM leading to metabolites with different anti-oestrogenic potential (Murphy et al, 1990).
– Loss or mutations of the oestrogen receptor, or of the oestrogen responsive element (ORE) of DNA (Mahfoudi et al, 1995; Murphy et al, 1996).
– Post-translational modifications of the OR (Denton et al, 1992).
– Involvement of different co-factors in transcriptional machinery (Landel et al, 1994; Lavinsky et al, 1998).
– Conformational changes of the secondary structure of the OR protein (Brzozowski et al, 1997; Maalouf et al, 1998).
– Interaction of (anti-) hormones with different OR-subtypes, OR α and β, leading to different transcription properties (Paech et al, 1997; Barkhem et al, 1998).
– Expression of specific breast cancer anti-oestrogen resistance genes (BCAR) (van Agthoven et al, 1998).

Mechanisms of anti-oestrogen resistance are mainly studied in established cell lines whose resistance was developed by in vitro treatment with increasing drug doses and selection of clones being able to grow in culture in the presence of anti-oestrogens (overview Lykkesfeldt, 1996; Katzenellenbogen et al, 1997). Culture conditions cannot completely mimic the in vivo environment and dynamic regulation mechanisms working in intact individuals, but probably select certain specific mechanisms of cell proliferation and resistance. We decided to develop a TAM-resistant subline of an originally very sensitive breast carcinoma xenograft (3366) by in vivo treatment of tumour-bearing nude mice with increasing doses of the anti-oestrogen. This procedure was performed during passaging of the xenograft over a period of 3 years. The established TAM-resistant subline (3366/TAM) was characterized according to histology and growth, cytostatic and anti-oestrogen resistance, expression of hormone receptors and hormone receptor dependent genes.

*Received 9 September 1999*
*Revised 13 January 2000*
*Accepted 18 January 2000*

*Correspondence to:* I Fichtner

### MATERIALS AND METHODS

### Animals

Six to eight female nude mice (Bom: NMRI-nu/nu) /group, aged 4–6 weeks and weighing 20–24 g, were used for the experiments. The conditions of breeding and maintenance of the animals have already been described (Naundorf and Arnold, 1981). All animal experiments were performed according to the UKCCR Guidelines for the Welfare of Animals in Experimental Neoplasia and with permittance of the local responsible authorities (G V247/98).

### Tumour transplantation

The subcutaneous (s.c.) transplantation of the tumour pieces (size 4 × 4 mm) was carried out into a prepared pocket of the left flank region of nude mice anaesthetized with Radenarkon (40 mg $kg^{-1}$ i.p. Etomidat, Asta Medica, Frankfurt, Germany). The tumour diameters were measured once weekly using a caliper-like mechanical instrument and tumour volume (V) was calculated according to the formula $V = (\text{length} \times \text{width}^2)\ 2^{-1}$. The median volumes of each group were related to the initial value (relative tumour volume, RTV). In all experiments, if not otherwise mentioned, tumour-bearing mice received an oestradiol supplementation (oestradiol valeriate, 0.5 mg $kg^{-1}$ once a week i.m.). This supplementation leads to a physiological level of serum oestradiol (25–984 pg $ml^{-1}$) comparable with the human situation (25–600 pg $ml^{-1}$ in dependence on follicular phase).

### Development of TAM-resistant mammary carcinoma 3366/TAM

The newly established TAM-resistant mammary carcinoma originated from the sensitive parental breast tumour 3366 described by Naundorf et al (1992). The breast carcinoma-bearing nude mice were treated with increasing doses of TAM (1–50 mg $kg^{-1}$) over a period of 3 years until loss of TAM sensitivity in vivo. Then tumour-bearing nude mice were treated regularly once a week with 50 mg $kg^{-1}$ TAM during passages, except the last passage before an experiment.

### Substances

The following substances were used: 17β-oestradiol valeriate, E2D (Jenapharm, Jena, Germany), Tamoxifen (Sigma, Chemie GmbH, Germany), ICI 182 780 (gift of Zeneca Pharmaceutical, Macclesfield, UK).

### Histological investigations

The histological procedures were carried out using routine methods. For the histological preparation 5% formalin was used as fixative and the sections of the tumour preparations were stained with haematoxylin/eosin.

### Receptor determination

The oestrogen (OR) and progesterone receptors (PR) in tumour tissue were determined according to the Abbott OR and PR enzyme immunoassay instructions (Abbott ER-EIA Monoclonal, Abbott GmbH Wiesbaden, Germany). The cut-off value for a positive receptor status for OR and PR is >15 fmol $mg^{-1}$ cytosol protein. The protein content of the cytosol fraction was determined according to the method of Lowry et al 1951.

### Nucleotide sequence determination of the hormone binding domain

The examined breast tumour specimens were shock-frozen in liquid nitrogen and stored at –80°C. Total RNA was isolated by the method of guanidinium thiocyanate extraction using a reagent system supplied by Promega (RNAgents, Promega Corporation, Madison, USA). The isolated RNA was resuspended in 1 × TE-buffer (10 mM Tris HCl pH 8.0, 1 mM EDTA), quantified spectrophotometrically and stored in aliquots at –80°C until used. RT and PCR reactions were carried out using thermostable rtTh-polymerase and appropriate rtTh PCR reagents (GeneAmp rtTh-RNA-PCR-System, Perkin Elmer Corporation, Foster City, USA) according to the general protocol supplied by the manufacturer. 200 ng of total RNA were reverse-transcribed in a final volume of 20 μl containing 200 μM dNTP's, 1 mM $MnCl_2$, 5 units rtTh-polymerase and 800 nM of the specific 3′-primer (3 min at 90°C followed by 15 min at 65°C). The subsequent cDNA amplification was performed in a final volume of 100 μl containing the whole RT reaction mixture, chelating buffer, 1 mM $MgCl_2$ and 160 nM of 5′-primer. The thermal profile of the amplification reaction involved an initial 2 min denaturation step at 95°C followed by 35 cycles of denaturation at 94°C (1 min) and annealing/extension at 64°C (1 min) and a final elongation at 72°C for 10 min. The selected pair of primers used in RT-PCR encompassed a 634 bp fragment in the hormon binding region of OR mRNA: hERb5′: GGC TTA CTG ACC AAC CTG GCA G (pos. 1390–1411); hERb3′: ACG GCT AGT GGG CGC ATG TAG G (pos. 2004–2025) (positions refer to GenBank No. X03635). After cleaning up the PCR-amplified fragment by agarose gel electrophoresis and extraction with QiaQuick Kit (Qiagen Inc., Chatsworth, USA) the sequence determination was carried out by InViTek Gmbh (Berlin-Buch) using a cycle sequencing protocol and an ABI-PRISM automated sequence analyser (Applied Biosystemes Inc., Foster City, USA). Both strands were sequenced.

### Expression of oestrogen receptor regulated genes

The oestrogen-regulated genes pS2 and cathepsin D were determined by Northern analysis. Frozen tissues were homogenized in a microdismembrator with pre-cooled (–180°C) chambers. TRIzol®Reagent (Life Technologies), 1 ml per 100 mg tissue, was added, and total RNA isolated as recommended by the supplier. Poly $(A)^+$RNA was isolated with the Oligo $(dT)_{25}$ coupled magnetic beads (Dynal, Oslo, Norway) according to the manufacturer's manual. Two micrograms of poly$(A)^+$RNA were denatured with glyoxal/DMSO solution, run on a 1.2% agarose gel and transferred to a nylon membrane (Nytran 13N, Schleicher & Schuell, Dassel, Germany). The probe used for Northern hybridization to pS2 was *Eco*RI lineriazed pS2 (Masiakowski et al, 1982), the probe for cathepsin D was oligonucleotide 5′-TTAACGTAGGT-GCTGGACTTGTCGCTGTTGTACTT-3′ (Augereau et al, 1988). The 1.1 kb *Pst*I fragment of 36B4 from MCF-7 cloned into the pBR322 vector (Laborda, 1991) was used as a control for loading. The plasmid-derived probes were labelled with [α-$^{32}$P]-dCTP (Amersham, Aylesbury, UK) using the Megaprime DNA labelling

© 2000 Cancer Research Campaign kit (Amersham) to a specific activity of $1–2 \times 10^9$ dpm $\mu g^{-1}$ DNA and Northern blots were hybridized as described (Madsen et al, 1992). The oligonucleotide probe was end-labelled with [$\gamma$-$^{32}P$]-ATP (Amersham) to a specific activity of $0.5–2 \times 10^7$ dpm $pmol^{-1}$ using T4 polynucleotide kinase (BRL). Northern blots were hybridized as described (Lykkesfeldt et al, 1994). The blots were exposed to Kodak X-OMAT AR-5 films at –80°C. For quantification, the blots were exposed to a PhosphorImager screen (Molecular Dynamics, Sunnyvale, CA, USA) for 1–5 days and the ImageQuant software was used for the calculations.

### RESULTS

### Histological characterization of the human mammary carcinomas 3366 and 3366/TAM

Histological studies of the sensitive breast carcinoma 3366 revealed a solid ductal invasive mammary carcinoma with moderate differentiation (Figure 1A). After treatment of tumour bearing nude mice with 0.5 mg $kg^{-1}$ 17β-oestradiol for 4 weeks, histological pattern of tumour changed to an 80% duct-forming growth (Figure 1B), whereas in the untreated tumour the solid growth prevailed. When the tamoxifen-resistant subline was treated with oestrogen, the histological appearance did not change, in comparison to the untreated control (Figures 1C and 1D).

### Growth behaviour of mammary carcinomas 3366 and 3366/TAM

As Figure 2 shows, the growth of the breast carcinoma 3366 in nude mice was strongly dependent on the supplementation with oestrogen. While the saline-treated tumours remained small in size (4–5 mm diameter), a treatment of mice with 0.5 mg $kg^{-1}$ oestradiol valeriate once a week during the experimental period of 10 weeks led to an approximately 20-fold increase of tumour volume. The anti-oestrogens TAM and ICI 182 780 completely prevented the growth of the xenografts, even when combined with oestradiol. Similar therapeutic effects in the 3366 xenografts were also obtained, when the tumours were transplanted to oestradiol supplemented male or ovariectomized female nude mice, and TAM was also active following oral or intraperitoneal routes of administration (data not shown). The TAM-resistant subline 3366/TAM (Figure 3) is similarly stimulated in growth by oestradiol. The anti-oestrogens TAM and ICI 182 780 administered to oestrogen supplemented mice are not able to significantly prevent it. In the experiment presented, TAM and ICI treatment of mice without oestradiol supplementation led to a growth similar to the solvent-treated tumours, while in three of seven experiments TAM stimulated growth (data not shown), indicating its partial oestrogenic properties. The TAM-resistance was persistent for up to six tumour passages without TAM treatment of nude mice.

### Hormone receptor expression

Both the original xenograft 3366 and the TAM-resistant subline express OR (113 ± 64 or 194 ± 55 fmol $mg^{-1}$ protein, respectively, obtained as mean ± standard deviation from 5–8 tumours each) and can therefore be considered as being OR positive. When tumour-bearing mice were treated for 6 weeks with 17β-oestradiol valeriate (E2D) and the xenografts were harvested at several days after last treatment, a regulation of OR expression became evident (Figure 4). In the breast carcinoma 3366 a short period of

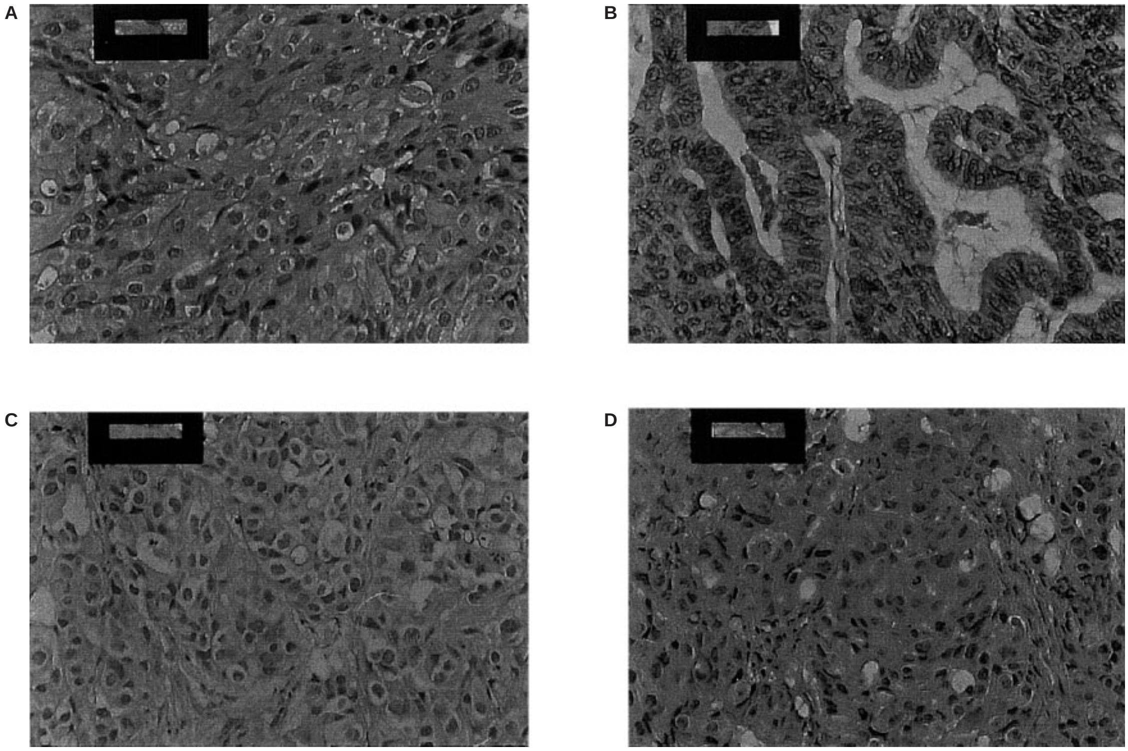

**Figure 1** Histology of breast cancer xenografts. (**A**) 3366, untreated; (**B**) 3366 treated for 4 weeks with 17β-oestradiol valeriate (0.5 mg $kg^{-1}$ once a week i.m.); (**C**) 3366/TAM untreated; (**D**) 3366/TAM, treated for 4 weeks with 17β-oestradiol valeriate. Magnification × 150

 *© 2000 Cancer Research Campaign*

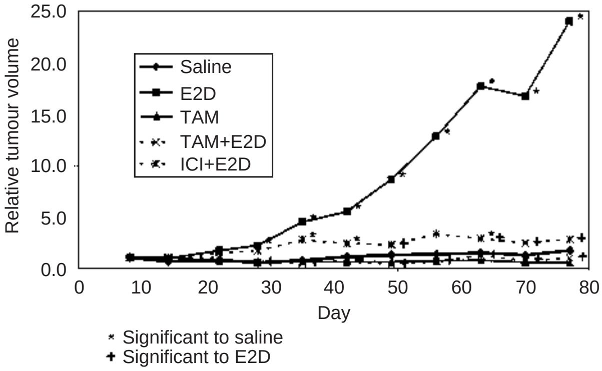

**Figure 2** Growth of breast carcinoma 3366 in nude mice (six per group) during treatment with saline, oestradiol valeriate (E2D, 0.5 mg kg$^{-1}$ once a week i.m.), TAM (50 mg kg$^{-1}$ twice a week i.m.) or ICI 182 780 (25 mg kg$^{-1}$ twice a week i.m.). Treatment was initiated 1 week after tumour transplantation (fragment of 2–3 mm diameter, s.c.) and continued until the end of the experiment

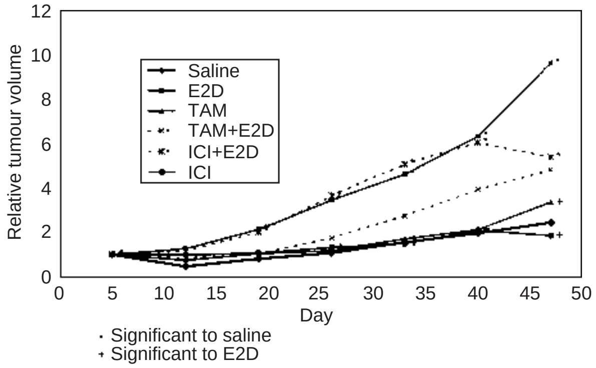

**Figure 3** Growth of breast carcinoma 3366/TAM in nude mice (six per group). For treatment schedules and doses see Figure 2

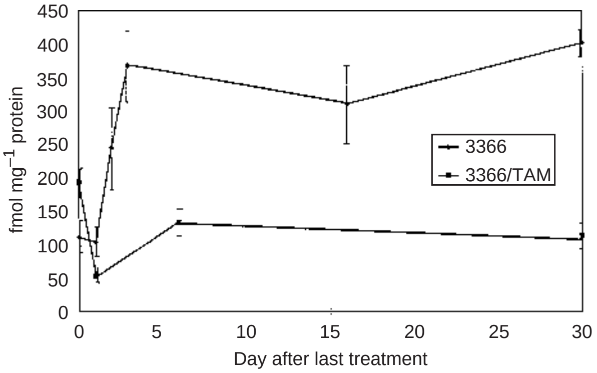

**Figure 4** Regulation of OR expression after cessation of oestradiol treatment (0.5 mg kg$^{-1}$ 17β-oestradiol valeriate once a week i.m. for 6 weeks). Each value represents the mean ± standard deviation from 5–8 tumours each decreased levels (day 1) is followed by a significant upregulation, lasting for more than 30 days after last E2D administration. In contrast, the TAM-resistant tumours never expressed higher than initial values after the finish of therapy. These results indicate difference in the dynamics of OR expression regulation following cessation of hormonal treatments between TAM-sensitive and -resistant breast carcinomas. Both the TAM-sensitive and the -resistant line expressed equally low levels of the ORβ in an immunohistochemical assay (data not shown).

The PR protein was expressed at 9 ± 8 or 15 ± 16 fmol mg$^{-1}$ protein (mean ± standard deviation from 5–8 samples each) in the TAM-sensitive or -resistant line, respectively. A treatment of in vivo tumours with E2D for several weeks enhanced expression significantly to 287 ± 104 or 419 ± 358 fmol mg$^{-1}$, a treatment with TAM to 84 ± 2 or 77 ± 26 fmol mg$^{-1}$. This induction of PR expression suggests a comparable functioning of transcription machinery independent of a response of the tumours to (anti-) hormonal treatment.

### Sequence comparison of the hormone-binding domain of TAM-sensitive and -resistant mammary carcinoma 3366

In order to evaluate whether the change in sensitivity of TAM could be attributed to a mutated HBD of the OR, we undertook a comparative sequence analysis of this region in both the parental tumour line 3366 and the TAM-resistant subline. The nucleotide sequence determination was performed by cycle sequencing of an RT-PCR-amplified 634 bp-fragment encompassing the pos. 1390–2026 (GenBank No X03635) (Green et al, 1986) in the hormone-binding region of OR-mRNA. The analysed sequences of both tumour lines – the TAM-sensitive and the -resistant one – were shown to be absolutely identical. No mutations could be detected, indicating that no sequence change in the HBD is responsible for the established TAM-resistance of the mammary carcinoma line 3366. Furthermore, no amino acid replacement in helix 12 (Brzozowski et al, 1997; Maalouf et al, 1998) and the transcriptional activation region 2 (TAF-2) described by others (Montano et al, 1995) could be found that might lead to agonistic activities of TAM. The determined sequences differ only in position 1559 (GenBank No X03635) from other published human OR sequences (GenBank No X03635, M12674, I08538), which leads to an amino acid substitution from valin to glycine in the deduced protein sequence. Furthermore, in both tumour lines a coexpression of an exon 7 splice variant of OR-mRNA could be detected which was also identified by sequencing. The sequence data of the HBD of mammary carcinoma line 3366 are available from EMBL Nucleotide Sequence Database (EBI, Hinxton, Cambridge, UK) under accession number Z75126.

### Expression of oestrogen receptor regulated genes

Gene expression of the two oestrogen regulated genes pS2 and cathepsin D have been normalized to the ribosomal protein 36B4, and in Figure 5 the mRNA levels are presented relative to the level of the respective gene in MCF-7 cells. The pS2 expression is extremely low in the 3366 tumour, about 33- to 45-fold lower in control 3366 tumours than in MCF-7 cells (Figure 5A). 3366 tumours from animals treated with oestradiol have a pS2 expression level as in tumours from animals without treatment. Treatment with tamoxifen resulted in a decreased expression level of pS2 to about 40% of the level in tumours from untreated animals. The total group of tamoxifen resistant 3366/TAM tumours have a significantly higher pS2 expression level than the total group of 3366 tumours ($P < 0.002$, Mann Whitney U-test, two-tailed). Treatment

*© 2000 Cancer Research Campaign* 

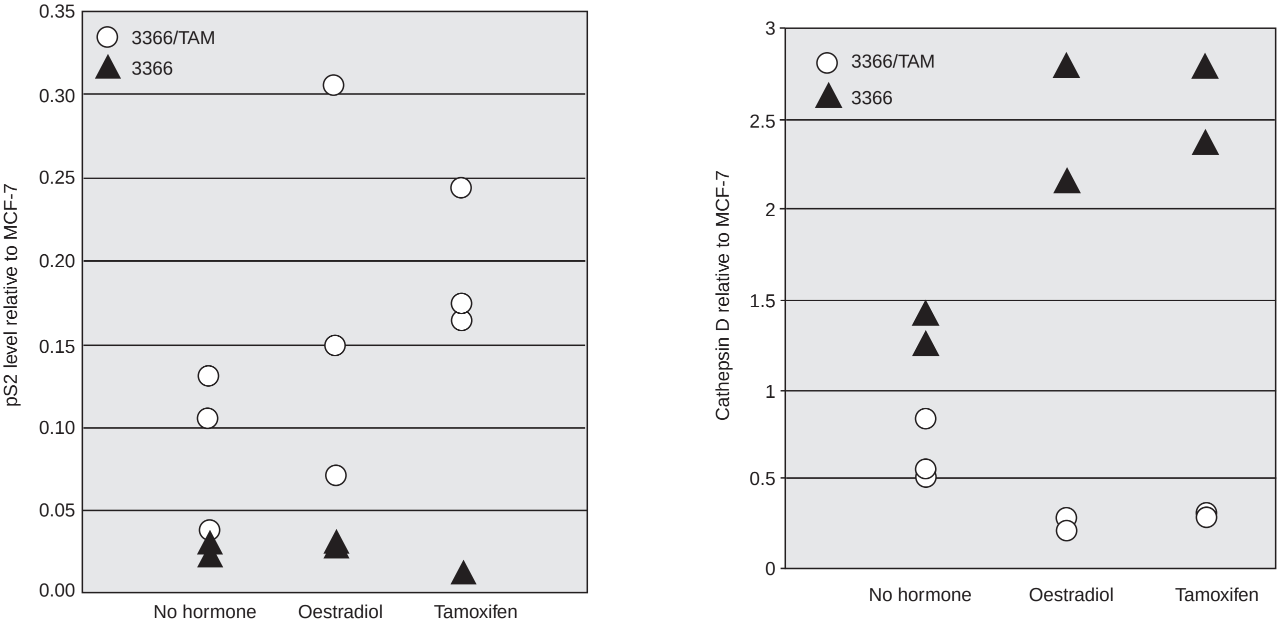

**Figure 5** Phosphorimager scan data showing the levels of pS2 (**A**) and cathepsin D mRNAs (**B**) in 3366 and 3366/TAM tumours relative to the level in MCF-7 cells. Animals received either no hormone treatment, treatment with oestradiol or treatment with tamoxifen. Poly $(A)^{+}$RNA was prepared from homogenized tumour tissue or MCF-7 cell pellets. The RNA was run on an agarose gel, blotted onto a nylon membrane and the amount of transcripts of pS2, cathepsin D and 36B4 were determined by hybridization with randomly labelled probes. The 36B4 mRNA was used as an internal control and the Phosphorimager scan values were normalized to the amount of this transcript before expressed relative to the level in MCF-7 cells.

with oestradiol or tamoxifen had no significant effect on pS2 expression in 3366/TAM tumours. The cathepsin D expression level in control 3366 tumours was in the same order of magnitude as in MCF-7 cells (Figure 5B). 3366 tumours from both oestradiol and tamoxifen treated animals had higher cathepsin D levels than in tumours from animals without treatment. Cathepsin D expression levels in control 3366/TAM tumours were also close to the level in MCF-7 cells. However, in these tumours, both oestradiol and tamoxifen reduced the level of cathepsin D expression.

### DISCUSSION

Endocrine therapy of breast carcinoma is an established procedure, especially for hormone receptor-positive cancers. Unfortunately, about one third of the receptor positive patients originally fail to respond to, for example, the anti-oestrogen tamoxifen, and another large majority of tumours eventually develop acquired resistance. Though several hypotheses exist on the mechanism of anti-oestrogen resistance, the ultimate reasons still remain unclear (reviews Osborne and Fuqua, 1994; Tonetti and Jordan, 1995; Lykkesfeldt, 1996).

Investigations concerning the response of cancer cells to anti-oestrogens mainly utilize molecular approaches or cell lines with in vitro selection for anti-oestrogen independence (review Lykkesfeldt, 1997). Only few literature reports refer to in vivo studies. Osborne et al (1987) and Gottardis and Jordan (1988) observed an inhibited tumour growth of the MCF-7 line in nude mice upon oestradiol withdrawal with or without tamoxifen. After 3–4 months of endocrine therapy, tumours started to regrow in a hormone independent way, though receptor analyses showed maintained OR- and PR-levels. Later on it was shown in the same model (Wolf and Jordan, 1994) that an inverse relation between OR and epidermal growth factor receptor levels existed and that the $E_2$-induced PR expression was almost completely abolished. Another group (Thompson et al, 1993) reported on an oestrogen-independent variant of the MCF-7 line forming locally invasive structures after transplantation into the mammary fat pad. This feature was accompanied by elevated levels of the oestrogen-inducible cathepsin D.

These relatively few literature reports on in vivo models of hormonal resistance exclusively utilized the MCF-7 model. Considering the few available OR-positive breast cancer cell lines (Clarke, 1996) we developed an in vivo model in a relatively clinically related manner. Starting from the very TAM-sensitive, OR-positive mammary carcinoma 3366, derived from a ductal invasive carcinoma of a menopausal patient (Naundorf et al, 1992), we developed a TAM-resistant subline by subsequent treatment of tumour-bearing nude mice during passaging with increasing doses of TAM. After about 3 years the resistance phenotype was obvious, resulting in a complete lack of remissions to TAM as was seen in the parental line. As both lines failed to grow in vitro, further comparative characterization was exclusively performed with the in vivo growing xenografts.

### Oestrogen receptor expression

Both lines have to be considered as OR-positive, both concerning the α- and the β-subtype of the protein. This observation coincides with literature reports on TAM-resistant sublines of MCF-7 (Gottardis and Jordan, 1988; Osborne et al, 1987; Lykkesfeldt et al, 1994; Madsen et al, 1997) also maintaining their ORα-positivity despite anti-oestrogen resistance. Also, it was clinically reported that in 69% of TAM-resistant tumours the initial OR-level was maintained (Johnston et al, 1995).

*© 2000 Cancer Research Campaign*

### Oestrogen receptor regulation

Despite the relatively similar absolute values of OR in the TAM-sensitive and -resistant sublines, a distinct difference in the dynamics of expression levels after several weeks' cessation of hormone treatment became evident. While in the parental 3366 tumour a transient downregulation (after 1 day) was followed by a long-standing upregulation (up to 30 days), such an enhancement was absent in the 3366/TAM subline. This observation can also be interpreted as a 'normalizing' reaction following the probable downregulation of OR during an oestradiol treatment occurring in the sensitive, but not the resistant, line.

On the other hand, it has to be kept in mind that in the standard Abbott EIA a low salt extraction is used for cytosol preparation. This means that hormone-bound receptors are not released from the chromatin and only the free receptors are determined. If this is the case, it is conceivable that a withdrawal of oestradiol treatment will, upon unchanged OR regulation, also give rise to an increase in free OR content as no hormone is available for binding to the ORs. However, this hypothesis delivers no explanation for the difference in regulation kinetics between the sensitive and the resistant line.

So far, OR regulation studies have been reported after short-term (24–48 h) incubation of receptor-positive cell lines with oestrogen or different anti-oestrogens (Pink et al, 1996; Martin et al, 1994; Jensen et al, 1999). These results can hardly be compared with our long-term in vivo effects, but they strengthen the fact that the OR expression, both on the mRNA and on the protein level, is strongly dependent on exogenous and endogenous factors and very dynamically regulated.

Also in a clinical study with breast tumour biopsies (Noguchi et al, 1993) an upregulation of OR and PR levels following an 8 day TAM treatment was demonstrated, but not correlated with the TAM response. Another clinical study evaluating breast cancer OR and PR levels in 2933 cases (Montella et al, 1996) revealed that OR positivity was more prevalent in tumours with lobular histology. This coincides with our observation that, following an oestradiol treatment of 3366 xenografts, with the proved increase in OR level a distinct change of histology to a marked lobular structure was induced. This apparent increase in differentiation was accompanied by a higher growth rate of the tumours and is probably a prerequisite for a TAM-response, as the lack of differentiation induction in the resistant line suggests.

### Oestrogen receptor structure

The comparative sequence analysis of the hormone binding domain of the OR isolated from 3366 or 3366/TAM xenografts revealed them to be absolutely identical. Both lines expressed both the wild-type fragment and the splice variant 7 in similar quantities. Though a large amount of molecular studies using artificial expression systems with a broad variety of OR mutants and variants have been documented, Tonetti and Jordan (1997) concluded in a review that at present, 'no compelling evidence suggests that mutation of the OR is the molecular mechanism producing TAM-stimulated growth in human breast cancer'.

This opinion and our results agree with literature reports (Karnik et al, 1994) documenting mutations of the OR in only two of 20 TAM-resistant clinical cancers. In addition, Madsen et al (1997) denied a relation of OR mRNA splice variants to anti-oestrogen resistance of MCF-7 sublines.

### Oestrogen receptor dependent gene and protein expression

The pS2 mRNA was discovered as the most abundant oestrogen-regulated mRNA in the human breast cancer cell line MCF-7 (Masiakowski et al, 1982). In human breast tumours, pS2 expression is correlated to OR expression and is a marker of oestrogen-dependent breast cancer (Rio et al, 1987; Foekens et al, 1994). The human 3366 tumour expresses pS2 mRNA, although at a very low level compared to MCF-7 cells. Treatment with oestradiol has no stimulatory effect on pS2 expression. This may be due to presence of low oestrogen levels in untreated animals. Tamoxifen acts as an oestrogen antagonist with respect to pS2 expression in 3366 tumour cells, and this is in concert with the antagonistic activity of tamoxifen on pS2 expression in MCF-7 cells (Westley et al, 1984; Lykkesfeldt et al, 1994). Cathepsin D was the first oestrogen-regulated protein secreted from MCF-7 cells to be described (Westley and Rochefort, 1980). In the 3366 tumour the cathepsin D mRNA expression level is comparable to the level in MCF-7 cells. A small induction of cathepsin D mRNA expression is observed in tumours from animals treated with oestradiol or with tamoxifen. This agonistic effect of both oestradiol and tamoxifen has also been seen in MCF-7 cells (Lykkesfeldt et al, 1994). Thus, in the OR-positive 3366 established from a human breast tumour sample, pS2 and cathepsin D regulation is similar to MCF-7 cells.

The tamoxifen-resistant 3366/TAM tumours appear to have an increased pS2 expression level compared to the 3366 tumours. Increased basal pS2 expression has also been observed in the tamoxifen resistant MCF-7/TAM$^{R}$-1 cells (Lykkesfeldt et al, 1994) and in ICI 182 780 resistant MCF-7 cell lines (Larsen et al, 1997). Tamoxifen does not downregulate pS2 expression in 3366/TAM tumours as in 3366 tumours, indicating loss of effect of tamoxifen on regulation of this gene. With respect to regulation of cathepsin D expression, both oestradiol and tamoxifen act as antagonists in the tamoxifen-resistant tumours 3366/TAM. These data indicate a change in the response to oestrogen and anti-oestrogen in the tamoxifen-resistant tumours and studies elucidating the underlying causes for these changed responses may provide important new information on OR-mediated gene expression.

In summary, the newly developed TAM-resistant subline of an OR-positive breast carcinoma represents a clinically relevant model for studying both mechanisms of anti-oestrogen resistance and for testing approaches to prevent or to overcome this phenotype. We show that neither absolute levels of OR expression nor mutations in the HBD are associated with TAM-resistance, but that the ability to regulate OR and oestrogen-responsive gene expression is apparently linked with the potential to respond to an anti-oestrogen treatment. Further studies to elucidate other probable mechanisms of TAM-resistance, like involvement of oestrogen receptor- or transcription-associated cofactors, are underway utilizing the in vivo model described here.

### ACKNOWLEDGEMENTS

The investigations were supported by a grant of the German Cancer Society (Dr Mildred-Scheel-Stiftung). The excellent technical assistance of M Becker, M Lemm and I Heiberg is gratefully acknowledged.

© 2000 Cancer Research Campaign

### REFERENCES

Augereau P, Garcia M, Mattei MG, Cavailles V, Depadova F, Derocq D, Capony F, Ferrara P and Rochefort H (1988) Cloning and sequencing of the 52K cathepsin D complementary deoxyribonucleic acid of MCF-7 breast cancer cells and mapping on chromosome 11. *Mol Endocrinol* **2**: 186–192

Barkhem T, Carlsson B, Nilsson Y, Enmark E, Gustafsson JA and Nilsson S (1998) Differential response of oestrogen receptor α and oestrogen receptor β to partial oestrogen agonists/antagonists. *Mol Pharmacol* **54**: 105–112

Brzozowski AM, Pike ACW, Dauter Z, Hubbart RE, Bonn T, Engström O, Öhman L, Green GL, Gustafsson JA and Carlquist M (1997) Molecular basis of agonism and antagonism in the oestrogen receptor. *Nature* **389**: 753–758

Clarke R (1996) Human breast cancer cell line xenografts as models of breast cancer – The immunobiologies of recipient mice and the characteristics of several tumorigenic cell lines. *Breast Cancer Res Treat* **39**: 69–86

Denton RR, Koszewski NJ and Notides AC (1992) Oestrogen receptor phosphorylation. Hormonal dependence and consequence on specific DNA binding. *J Biol Chem* **267**: 7263–7268

Early Breast Cancer Trial Collaborative Group (1992) Systemic treatment of early breast cancer by hormonal, cytotoxic, or immune therapy: 133 randomized trials involving 31 000 recurrences and 24 000 deaths among 75 000 women. *Lancet* **339**: 1–15, 71–85

Foekens JA, Portengen H, Look MP, van Putten WLJ, Thirion B, Bontenbal M and Klijn JGM (1994) Relationship of PS2 with response to tamoxifen therapy in patients with recurrent breast cancer. *Br J Cancer* **70**: 1217–1223

Gottardis MM and Jordan VC (1988) Development of tamoxifen-stimulated growth of MCF-7 tumors in athymic mice after long-term anti-oestrogen administration. *Cancer Res* **48**: 5183–5187

Green S, Walter P, Kumar V, Krust A, Bornert JM, Argos P and Chambon P (1986) Human oestrogen receptor cDNA: sequence, expression and homology to v-erb-A. *Nature* **320**: 134–139

Jensen BL, Skouv J, Lundholt BK and Lykkesfeldt AE (1999) Differential regulation of specific genes in MCF-7 and the ICI 182780-resistant cell line MCF-7/182R-6. *Br J Cancer* **79**: 386–392

Johnston SRD, Saccani-Jotti G, Smith IE, Salter J, Newby J, Coppen M, Ebbs SR and Dowsett M (1995) Changes in oestrogen receptor, progesterone receptor, and pS2 expression in tamoxifen-resistant human breast cancer. *Cancer Res* **55**: 3331–3338

Karnik PS, Julkarni S, Liu XP, Budd T and Bukowski RM (1994) Oestrogen receptor mutations in tamoxifen-resistant breast cancer. *Cancer Res* **54**: 349–353

Katzenellenbogen BS, Montano MM, Ekena K, Herman ME and McInerney EM (1997) Anti-oestrogens: mechanisms of action and resistance in breast cancer. *Breast Cancer Res Treat* **44**: 23–38

Landel CC, Kushner PJ and Greene GL (1994) The interaction of human oestrogen receptor with DNA is modulated by receptor-associated proteins. *Mol Endocrinol* **8**: 1407–1419

Laborda J (1991) 36B4 cDNA used as an estradiol-independent mRNA control is the cDNA for human acidic ribosomal phosphoprotein PO. *Nucleic Acids Res* **19**: 3998

Larsen SS, Madsen MW, Jensen BL and Lykkesfeldt AE (1997) Resistance of human breast-cancer cells to the pure steroidal anti-estrogen ICI 182,780 is not associated with a general loss of estrogen-receptor expression or lack of estrogen responsiveness. *Int J Cancer* **72**: 1129–1136

Lavinsky RM, Jepsen K, Heinzel T, Torchia J, Mullen TM, Schiff R, Del Rio AL, Ricote M, Ngo S, Gemsch J, Hilsenbeck SG, Osborne CK, Glass CK, Rosenfeld MG and Rose DW (1998) Diverse signaling pathways modulate nuclear receptor recruitment of N-CoR and SMRT complexes. *Proc Natl Acad Sci USA* **95**: 2920–2925

Lowry OH, Rosebrough NJ, Farr AL and Randall RJ (1951) Protein measurement with the folin phenol reagent. *J Biol Chem* **193**: 265–275

Lykkesfeldt AE, Madsen MW and Briand P (1994) Altered expression of estrogen-regulated genes in a tamoxifen-resistant and ICI 164,384 and ICI 182,780 sensitive human breast cancer cell line, MCF-7/TAM$^R$-1. *Cancer Res* **54**: 1587–1595

Lykkesfeldt AE (1996) Mechanisms of tamoxifen resistance in the treatment of advanced breast cancer. *Acta Oncol* **5**(Suppl): 9–14

Lykkesfeldt AE (1997) *Growth regulation of human breast cancer cells by oestrogens and anti-oestrogens* ISBN-no: 87–90212–14–2

Maalouf GJ, Xu W, Smith TF and Mohr SC (1998) Homology model for the ligand-binding domain of the human estrogen receptor. *J Biomol Struct Dyn* **15**: 841–851

Madsen MW, Lykkesfeldt AE, Laursen I, Nielsen KV and Briand P (1992) Altered gene expression of c-myc, epidermal growth factor receptor, transforming growth factor-alpha, and c-erb-B2 in an immortalized human breast epithelial cell line, HMT-3522, is associated with decreased growth factor requirements. *Cancer Res* **52**: 1210–1217

Madsen MW, Reiter BE, Larson SS, Briand P and Lykkesfeldt, AE (1997) Estrogen receptor messenger RNA splice variants are not involved in antiestrogen resistance in sublines of MCF-7 human breast cancer cells. *Cancer Res* **57**: 585–589

Mahfoudi A, Roulet E, Dauvois S, Parker MG and Wahli W (1995) Specific mutations in the estrogen receptor change the properties of antiestrogens to full agonists. *Proc Natl Acad Sci USA* **92**: 4206–4210

Martin MB, Saceda M, Garcia-Morales P and Gottardis MM (1994) Regulation of estrogen receptor expression. *Breast Cancer Res Treat* **31**: 183–189

Masiakowski P, Breathnach R, Bloch J, Gannon F, Krust A and Chambon P (1982) Cloning of cDNA sequences of hormone-regulated genes from the MCF-7 human breast cancer cell line. *Nucleic Acids Res* **10**: 7895–7903

Montano MM, Muller V, Trobaugh A and Katzenellenbogen BS (1995) The carboxy-terminal F domain of the human estrogen receptor: role in the transcriptional activity of the receptor and the effectiveness of antiestrogens as estrogen antagonists. *Mol Endocrinol* **9**: 814–825

Montella M, Biondi E, deMarco M, Cerra M, Cecco L, Botti G, Bonelli P and Tuccillo F (1996) Breast cancer estrogen and progesterone receptors: a multivariate analysis of associations with clinical and histologic characteristics in a series of 2933 consecutive cases. *International Journal of Oncology* **9**: 977–982

Murphy CS, Langan-Fahey SM, McCague R and Jordan VC (1990) Structure–function relationship of hydroxylated metabolites of tamoxifen that control the proliferation of estrogen-responsive T47D breast cancer cells in vivo. *Mol Pharmacol* **38**: 737–743

Murphy LC, Wang M, Coutt A and Dotzlaw H (1996) Novel mutations in the estrogen receptor messenger RNA in human breast cancers. *J Clin Endocrinol Metab* **81**: 1420–1427

Naundorf H and Arnold W (1981) Breeding of nude mice under improved conditions. *Zeitschrift fur Versuchstierkunde* **23**: 77–79

Naundorf H, Fichtner I, Büttner B and Frege J (1992) Establishment and characterization of a new human oestradiol – and progesterone-receptor-positive mammary carcinoma serially transplantable in nude mice. *J Cancer Res Clin Oncol* **119**: 35–40

Noguchi S, Motomura K, Inaji H, Imaoka S and Koyama H (1993) Up-regulation of estrogen receptor by tamoxifen in human breast cancer. *Cancer* **71**: 1266–1272

Osborne CK, Coronado EB and Robinson JP (1987) Human breast cancer in the athymic nude mouse: cytostatic effects of long-term antiestrogen therapy. *European Journal of Cancer and Clinical Oncology* **23**: 1189–1196

Osborne CK and Fuqua SAW (1994) Mechanisms of tamoxifen resistance. *Breast Cancer Res Treat* **32**: 49–55

Paech K, Webb P, Kuiper GGJM, Nilsson S, Gustafsson JA, Kushner PJ and Scanlan TS (1997) Differential ligand activation of oestrogen receptors ERα and ERβ at AP1 sites. *Science* **277**: 1508–1510

Pink JJ and Jordan VC (1996) Models of estrogen receptor regulation by oestrogens and antiestrogens in breast cancer cell lines. *Cancer Res* **56**: 2321–2330

Rio MC, Bellocq JP, Gairard B, Rasmussen UB, Krust A, Koehl C, Calderoli H, Schiff V, Renaud R and Chambon P (1987) Specific expression of the pS2 gene in subclasses of breast cancers in comparison with expression of the estrogen and progesterone receptors and the oncogene ERBB2. *Proc Natl Acad Sci USA* **84**: 9243–9247

Thompson EW, Brünner N, Torri J, Johnson MD, Boulay V, Wright A, Lippman ME, Steeg PS and Clarke R (1993) The invasive and metastatic properties of hormone-independent but hormone-responsive variants of MCF-7 human breast cancer cells. *Clin Exp Metastasis* **11**: 15–26

Tonetti DA and Jordan VC (1995) Possible mechanisms in the emergence of tamoxifen-resistant breast cancer. *Anticancer Drugs* **6**: 498–507

Tonetti DA and Jordan VC (1997) The role of estrogen receptor mutations in tamoxifen-stimulated breast cancer. *J Steroid Biochem Mol Biol* **62**: 119–128

van Agthoven T, van Agthoven TLA, Dekker A, van der Spek PJ, Vreede L and Dorssers LCJ (1998) Identification of BCAR3 by a random search for genes involved in antiestrogen resistance of human breast cancer cells. *EMBO J* **17**: 2799–2808

Westley B and Rochefort H (1980) A secreted glycoprotein induced by estrogen in human breast cancer cell lines. *Cell* **20**: 353–362

Westley B, May FEB, Brown AMC, Krust A, Chambon P, Lippman ME and Rochefort H (1984) Effects of antiestrogens on the estrogen-regulated pS2 RNA and the 52- and 160-kilodalton proteins in MCF-7 cells and two tamoxifen resistant sublines. *J Biol Chem* **259**: 10030–10035

Wolf DM and Jordan VC (1994) Characterization of tamoxifen stimulated MCF-7 tumor variants grown in athymic mice. *Breast Cancer Res Treat* **31**: 117–127

*© 2000 Cancer Research Campaign*
